# Engineering the next generation of CAR T- cells: precision modifications, logic gates and universal strategies to overcome exhaustion and tumor resistance

**DOI:** 10.3389/fonc.2025.1698442

**Published:** 2026-01-09

**Authors:** Juan Esteban Garcia-Robledo, Sergio Cabrera-Salcedo, Andreas Michael Brandauer, Francesco Romano, Joshua Rengifo-Martinez, Alejandro Toro-Pedroza, Juan Sebastián Victoria, Lady J. Rios-Serna, Alexandre Loukanov, Andrés Felipe Cardona, Pietro Genovese, Juan Camilo Baena

**Affiliations:** 1 LiliCAR-T Group, Fundación Valle del Lili, Cali, Colombia; 2 Centro de Investigación en Hematología y Oncología (CIHO), IDC Instituto de Cáncer Hemato Oncólogos, Cali, Colombia; 3 Faculty of Health Sciences, Universidad Icesi, Cali, Colombia; 4 Dana-Farber/Boston Children’s Cancer and Blood Disorder Center, Harvard Medical School, Boston, MA, United States; 5 Department of Pediatrics, Harvard Medical School, Boston, MA, United States; 6 Harvard Stem Cell Institute, Harvard University, Cambridge, MA, United States; 7 Clinical Research Center, Rio Grande Urology, El Paso, TX, United States; 8 Centro de Investigación en Reumatología, Autoinmunidad y Medicina Traslacional (CIRAT), Universidad Icesi, Cali, Colombia; 9 Department of Chemistry and Materials Science, National Institute of Technology, Gunma College, Maebashi, Japan; 10 Department of Oncology, Centro de Tratamiento e Investigación Sobre Cáncer Luis Carlos Sarmiento Angulo (CTIC), Bogotá, Colombia; 11 Department of Hematology/Oncology, Fundación Valle del Lili, Cali, Colombia

**Keywords:** Cell therapy 4, CAR T cells, solid tumors (ST), tumor microenvironment, genetic engineering

## Abstract

Chimeric antigen receptor (CAR) T-cell therapy has transformed the treatment landscape of hematologic malignancies, delivering durable remissions in diseases previously associated with poor outcomes. However, translating this success to solid tumors has proven challenging due to antigen heterogeneity, limited tumor infiltration, immunosuppressive tumor microenvironments, and progressive T-cell exhaustion. In response, next-generation CAR T-cell platforms have emerged that integrate advances in receptor architecture, intracellular signaling, and programmable control systems to enhance specificity, persistence, and safety. This review comprehensively examines recent innovations in CAR T-cell engineering, including optimization of extracellular binding domains, hinge and transmembrane modifications, fine-tuning of intracellular signaling motifs, and the incorporation of alternative protein scaffolds. We discuss logic-gated strategies such as synNotch receptors, inducible ON-switch CARs, inhibitory CARs, and modular adaptor systems that enable context-dependent activation and reduce off-tumor toxicity. In parallel, we explore approaches aimed at overcoming T-cell dysfunction through intrinsic checkpoint rewiring, cytokine armoring, and epigenetic reprogramming to sustain antitumor activity in hostile microenvironments. The development of allogeneic and off-the-shelf CAR T-cell products derived from healthy donors, induced pluripotent stem cells, natural killer cells, γδ T cells, and macrophages is also reviewed, highlighting strategies to mitigate graft-versus-host disease and host immune rejection while enabling scalable manufacturing. Finally, we address current translational bottlenecks related to immunogenicity, regulatory complexity, and production logistics, and outline future directions for integrating Boolean logic circuits, safety switches, and automated GMP-compliant processes. Collectively, these advances position next-generation CAR T-cell therapies as programmable and adaptable immunotherapeutic platforms with the potential to extend durable clinical benefit beyond hematologic cancers into solid tumors.

## 1 Introduction

Chimeric antigen receptor (CAR) T-cells are genetically engineered T lymphocytes modified to express a synthetic receptor that specifically recognizes tumor-associated or tumor-specific surface antigens in a TCR-independent manner, enabling precise recognition and attack of cancer cells, leading to targeted tumor cell elimination (1).

Since 2017, seven CAR T-cell products have been approved by the US Food and Drug Administration (FDA) for the treatment of B-cell acute lymphoblastic leukemia (B-ALL), diffuse large B-cell lymphoma (DLBCL), follicular lymphoma (FL), mantle cell lymphoma (MCL), chronic lymphocytic leukemia (CLL), and multiple myeloma (MM) (2). CAR T-cell therapy has significantly improved overall survival (OS) in patients with large B-cell lymphoma and progression-free survival (PFS) in patients with MM (3). Patients with FL and MCL have also achieved long-lasting remissions following CAR T-cell therapy (1).

The dramatic effectiveness of CAR T-cell therapy in blood cancers has made researchers and clinicians to consider its use on other conditions including solid tumors, infectious diseases, autoimmune disorders (4) and transplant-related graft-versus-host-disease (GvHD) (4, 5). In the field of solid tumors, conventional CAR T-cells have been shown to possess multiple weaknesses that have hindered their applications and effectiveness for tumor eradication. Most of these flaws are related to the inconsistent and heterogenous expression of the target antigen on cancer cells and a myriad of physical/chemical and molecular challenges imposed by the tumor microenvironment (TME), causing high rates of antigen escape, selection of cancer clones that do not express the target antigen and T-cell dysfunction (6). Therefore, preclinical studies and early clinical studies of conventional CAR T-cells on solid tumors have had disappointing results (7, 8).

To overcome these challenges and limitations, researchers have engineered multiple mechanisms to modify CAR T-cell behavior, making them suitable to navigate the TME and be effective in solid tumor elimination. The next generation of CAR T-cell therapies under development integrate innovative strategies to enhance T-cell potency, tumor heterogeneity, and improve current treatments by reducing its toxicity, thus broadening the pool of patients that can benefit from it. In this review we will cover some of the most promising advancements recently made in this field and their potential clinical applications.

## 2 The classic structure of CAR T-cells

The CAR consists of three essential domains: extracellular domain, transmembrane domain, and intracellular domain. The extracellular domain contains the antigen-binding moiety, which in most cases (including all currently FDA-approved therapies) may include a single-chain variable fragment (scFv) derived from an antibody and comprised of a variable heavy (VH) and light (VL) chains separated by a flexible linker (9). ScFvs sequences are taken from antibodies that have been designed to target a variety of cell surface molecules including proteins, carbohydrates, and glycolipids overexpressed by several malignancies which often display higher affinity than the canonical TCR, as is typical of high affinity scFvs; however, emerging evidence suggests that lower affinity CARs may actually enhance serial target engagement and reduce tonic signaling, thereby improving tumor clearance and persistence. The hinge domain, which links the scFv to the transmembrane region, is a key determinant of antigen accessibility and receptor flexibility. Its length and composition, typically derived from IgG1, IgG4, or CD8α sequences, influence the spatial configuration of the receptor and the efficiency of immune synapse formation. Optimization of hinge structure has been shown to enhance signaling potency and minimize steric hindrance, particularly when targeting membrane-proximal epitopes. Nevertheless, the binding domain can also contain complimentary binding peptides that are not derived from antibodies (10).

The transmembrane domain joins the extracellular domain by the hinge moiety, a polypeptide chain that gives flexibility and an increased reach to the binding domain, influencing the recognition of antigens that might reside closer to the membrane (11). The receptor transmembrane region provides support and stability to the plasma membrane (12). Most transmembrane domains are derived from transmembrane receptor proteins. The transmembrane polypeptide sequence influences the activating signal transmitted to the intracellular domain. Transmembrane domains are composed of either Fc regions of IgG1 immunoglobulins, Ig-like regions of the T-cell co-receptor CD4 or CD8, or costimulatory molecules like CD28. These domains have a crucial role in facilitating CAR-target interactions and further signaling (13, 14).

The intracellular domain is responsible for initiating the signaling cascade that leads to T-cell activation, proliferation, and cytotoxic activity against target cells. It consists of both signaling and co-stimulatory domains. The primary component of the intracellular domain in CAR T-cells therapy is the CD3z chain, which contains immunoreceptor tyrosine-based activation motifs (ITAM) essential for T-cell activation (15, 16). Upon antigen engagement by the CAR, it triggers a nuclear factor of the activated T cell (NFAT)-dependent transcriptional pathway. In addition to activating NFAT, CAR signaling also engages the NF-κB and AP-1 pathways. Together, these transcriptional programs regulate the expression of cytokines such as IL-2 and IFN-γ, cytolytic effectors including perforin and granzyme B, and co-stimulatory receptors essential for complete T cell activation, proliferation, and persistence. Signaling domains must be combined with costimulatory molecules, such as CD28, 4-1BB, CD27, inducible T cell co-stimulator (ICOS), and OX40, among others, to generate a complete activation of T-cell effector mechanisms (17).

Co-stimulatory molecules determine efficient receptor activation, the development of an immunological memory phenotype, the kinetics of anti-tumor response, cytotoxic function, and associated toxicities. The selection of a co-stimulatory domain significantly impacts the phenotype and metabolic signature of T-cells (17) (**Table 1**).

**Table 1:** Summary of major co-stimulatory domains used in CAR T-cell design.

Co-stimulatory domain	Key Pathways activated	Functional outcome	Product
CD28	PI3K–AKT–mTOR	Rapid effector function, glycolytic metabolism, short persistence	Yescarta
4-1BB (CD137)	NF-κB, p38-MAPK	Mitochondrial biogenesis, central memory phenotype, prolonged survival	Kymriah
ICOS	PI3K-independent, YMFM motif	Th17/Tfh polarization, high cytokine output	ICOS-B7H3-CAR (74)
OX40 (CD134)	TRAF2/NF-κB	Memory maintenance, reduced exhaustion	GD2-CAR.OX40.28.z.ICD9 (75)
CD27	TNFR-associated	Enhanced survival, reduced apoptosis	CBG-002 (76)

For instance, CD28 and 4-1BB confer distinct functional characteristics: CD28 promotes a highly potent but short-lived effector phenotype, characterized by strong cytolytic activity, high IL-2 secretion, and increased glycolysis. Additionally, CD28 signaling has been shown to support memory T-cell differentiation through mitochondrial remodeling and increased mitochondria respiratory capacity, enabling rapid response to secondary stimulation (18, 19). In contrast, 4-1BB enhances T cell expansion and persistence *in vivo*, supports oxidative metabolism, reduces susceptibility to exhaustion, and facilitates the generation of central memory CAR-T cells (20). 4-1BB co-stimulation actives signaling through p38-MAPK pathway, promoting enhanced mitochondrial biogenesis (21). This is associated with healthier T-cells with increased performance and metabolic flexibility within the TME, contributing to improved tumor control (22).

ICOS, a member of the immunoglobulin superfamily, is expressed at low levels on the surface of naïve T cells. It binds exclusively to its ligand, ICOS-L, which is found on B-cells, macrophages, and lung epithelial cells. ICOS primarily promotes the differentiation of T follicular helper (Tfh) cells, which are essential for B-cell affinity maturation within germinal centers (23). Additionally, ICOS plays a role in enhancing anti-tumor T-cell responses and contributes to the pathogenesis of graft-versus-host disease (GvHD) (24). The cytoplasmic signaling domain of ICOS closely resembles that of CD28, sharing a similar proximal YMFM motif. The incorporation of the ICOS signaling domain confers inherent multipotency and enhanced self-renewal capacity to CAR-T cells TH17 polarization, that observed in CAR-T cells containing CD28 or 4-1BB costimulatory domains. Also rapid signaling kinetics, grater phosphorylation intensity, greater cytokine release, rapid tumor regression, and persistence long-lived (2).

The incorporation of tumor necrosis factor receptor (TNFR) superfamily molecules such as CD27 and OX40 in CAR design supports improved antigen-dependent memory formation and promotes T cell survival. These TNFRs play a key role function to sustain the T cell response and drive signaling essential for memory T cell development. OX40 (CD134, TNFRSF4) expression is restricted to activated T-cells. It is critical for regulating clonal expansion through expression of pro-survival molecules and cell cycle regulators. Additionally, OX40 contributes to T-cell differentiation and maintenance of a memory T-cell pool following antigen exposure. It also indirectly regulates effector differentiation through modulation of cytokine production and cytokine receptor expression. CAR T cells expressing OX40 exhibit reduced susceptibility to exhaustion and demonstrate enhanced long-term persistence, and increased dependency on mitochondrial metabolism (25).

### 2.1 The different generations of CAR T-cells

To date, five generations of CAR T-cells have been developed, each designed to address specific limitations observed in earlier iterations. These generational advances reflect a progressive refinement of structure and function aimed at improving anti-tumor efficacy, persistence, safety, and adaptability in the tumor microenvironment (TME).

CAR T cell design has evolved through three major generations to improve antitumor-efficacy and persistence. First-generation CARs, developed by Zelig Eshhar and colleagues in the late 1980s, consisted of an extracellular scFv recognizing tumor-associated antigens, a hinge or spacer, and an intracellular CD3ζ signaling domain containing ITAMs. Although these constructs activated TCR signaling, the absence of costimulatory domains limited expansion, persistence, and clinical efficacy. Second-generation CARs, introduced in the late 1990s, incorporated a costimulatory domain such as CD28 or 4-1BB alongside CD3ζ, providing dual signaling that enhanced proliferation, interleukin-2 production, cytotoxicity, and *in vivo* persistence, leading to the clinical approval of tisagenlecleucel (Kymriah) and axicabtagene ciloleucel (Yescarta). Third-generation CARs combined two costimulatory domains, typically CD28 with 4-1BB or ICOS with OX40, to further potentiate activation and survival. Preclinical studies demonstrated superior expansion and tumor clearance compared with earlier constructs; however, clinical data confirming their superiority remain limited, and their incremental benefit over second-generation CARs continues to be evaluated. Consequently, the field has shifted its focus toward fourth and fifth generation CARs, which integrate cytokine payloads, signaling cassettes, and modular recognition platforms, aligning with the next decade’s translational goals in solid tumors.

Fourth-generation CAR T cells, also referred to as TRUCKs (T cells Redirected for Universal Cytokine-mediated Killing), are engineered to overcome key limitations such as poor infiltration, antigen heterogeneity, and the immunosuppressive TME. These CARs retain the core signaling architecture of second-generation CARs—comprising an scFv, CD3ζ, and a costimulatory domain—but also include an inducible transgene expression system. Typically, they are equipped with a nuclear factor of activated T cells (NFAT)-responsive promoter driving the expression of cytokines (e.g., IL-12) or other immunomodulatory factors. Upon antigen engagement, T cells not only initiate cytotoxic responses but also release transgenic products designed to modify the local immune environment, recruit additional immune cells, or counteract tumor-mediated suppression. This approach allows for a more flexible and context-dependent response and has shown promising results in preclinical models.

Fifth-generation CAR T cells represent a modular and potentially more versatile platform. A notable feature is the inclusion of a biotin-binding immunoreceptor (BBIR) or synthetic universal immune receptor (SUPRA) system. These designs decouple antigen recognition from intracellular signaling by using an extracellular domain that binds to biotinylated or tagged molecules, allowing for tunable and multiplexed targeting. Additionally, these CARs are compatible with low-antigen-density tumors or tumors expressing multiple heterogeneous targets. Some designs also integrate intracellular domains that can recruit endogenous cytokine signaling pathways, such as those involving JAK-STAT, thereby providing additional proliferative or survival cues upon activation. The modularity of fifth-generation CARs enables better control over specificity, intensity, and duration of the immune response and facilitates large-scale and standardized manufacturing.

In summary, each generation of CAR T-cells has built upon the limitations of its predecessors. First-generation CARs established the basic framework but lacked persistence and efficacy. Second-generation CARs introduced costimulatory domains, markedly improving function. Third-generation CARs attempted to enhance activation further with dual co-stimulation, though clinical benefits remain under investigation. Fourth-generation CARs incorporated inducible payloads to reshape the TME, and fifth-generation CARs introduced modular, tunable architectures to address target heterogeneity and enable scalable production. Together, these iterative advances reflect the ongoing efforts to optimize CAR T cell therapy for a broader range of cancers with improved safety and efficacy profiles.

## 3 Blueprints for better killers: structural innovations in CAR sequences

The TME poses significant challenges to effective cell therapy. Extensive and innovative research suggests that certain modifications in the architecture of the CAR sequence might optimize CAR T-cell performance, yielding it more suitable to solid tumor cytotoxicity.

### 3.1 Extracellular domain engineering

#### 3.1.1 Binding domain

Most currently FDA-approved CAR T-cell therapies use a scFv derived from the high-affinity murine hybridoma clone FMC63, which targets CD19 (26). However, scFv-based constructs present several limitations, including reduced protein stability and a tendency toward aggregation. These features can lead to tonic signaling, resulting in T-cell exhaustion and ultimately impairing both the short-term efficacy and long-term persistence of CAR T cells (27). As an alternative, nanobodies which are highly stable, monomeric antigen-binding domains derived from heavy-chain-only antibodies obtained from llamas and other camelids, offer several advantages (28).

These compact immunoglobulin G-like domains, approximately 110 amino acids in length, possess binding capabilities comparable to conventional monoclonal antibodies (29). Their inherent modularity facilitates seamless integration into CAR designs, while minimizing the risk of protein instability or loss of binding affinity (30). Indeed, ciltacabtagene autoleucel (cilta-cel), an anti-BCMA CAR T-cell therapy that uses nanobody binders, was approved by the FDA in February 2022 for relapsed/refractory MM (31).

Another alternative, the Designed Ankyrin Repeat Proteins (DARPins) are versatile synthetic proteins composed of multiple, stacked repeats of ankyrin protein domains, a typical configuration includes four to six repeats, engineered to mimic the binding properties of natural antibodies (32). DARPins exhibit inherent thermostability and high solubility, making them highly customizable for high-affinity binding to a broad range of protein targets (29). For example, it may aid in stabilizing CAR surface expression while reducing the propensity for CAR dimerization, a phenomenon that drives deleterious CAR tonic signaling. Currently, DARPins-based CARs against HIV-infected cells have proven to be a leading and robust targeting system, at least equivalent to efficacy of scFv-based CARs. DARPin CAR T-cells against HER2 also shown a similar efficacy *in vitro* to scFv CAR T-cell (33, 34).

Natural ligand-based CARs represent another design strategy in which endogenous human protein ligands are used as the antigen-recognition domain to engage cytotoxic effector cells. Compared to scFv-based constructs, this approach offers the advantage over scFv in their low potential immunogenicity, as the targeting moiety is derived directly from a natural human protein. This requires almost no protein re-engineering, simplifying the execution of CAR design. In addition, although the simplicity of natural ligand CAR design is appealing, the inability to readily modulate or tune natural ligands and receptors as CAR binders can also be a drawback if the physiological ligand-receptor interaction is insufficient, due to affinity, avidity, or otherwise, to drive CAR activation (35). Nonetheless, for targets with a well-defined and selective natural ligand, this strategy offers several compelling advantages, including reduced immunogenicity, preserved structural stability, and simplified CAR design.

Adnectins are derived from a single-domain scaffold based on the tenth type III domain of human fibronectin, which confers low immunogenic potential due to their fully human origin. One study demonstrated that CAR T-cells employing adnectin-based antigen recognition domains exhibit comparable tumor cell killing efficacy to those using conventional scFvs. These findings suggest that the use of adnectin scaffolds for CAR design is not only feasible but also holds significant promise for future CAR T-cell engineering efforts (36).

An emerging class of CAR binding domains includes fully synthetic protein elements, exemplified by the platform developed by the biotechnology company Arcellx. Their anti-BCMA CAR T-cell therapy, currently in phase I clinical trials (NCT04155749) for the treatment of R/R MM, employs a synthetic D-domain binding technology for tumor antigen recognition. These D-domains are composed of a three alpha-helical bundle, an engineered sequence variance for target specificity. As entirely synthetic constructs, D-domains offer potential advantages over conventional scFv-based binders, including improved structural stability, reduced aggregation, and minimized immunogenicity (37).

Adnectin and D-domain based CAR constructs are at distinct stages of translational development. Adnectin-derived CARs have demonstrated proof of concept efficacy in preclinical models of glioblastoma and ovarian cancer but have not yet entered clinical testing. Conversely, the D-domain platform developed by Arcellx (CART-ddBCMA) has advanced to phase 2 (NCT05396885) clinical evaluation in relapsed/refractory multiple myeloma, where early results indicate ORR of 95% with CR/sCR rate of 62%, demonstrating deep and durable efficacy and manageable safety in a high-risk 4L+ RRMM population including triple- and penta-class refractory disease. These outcomes demonstrate the feasibility of advancing fully synthetic antigen binders to clinical application, although questions regarding large scale manufacturing and long term immunogenicity require further evaluation (38).

**Figure 1:**
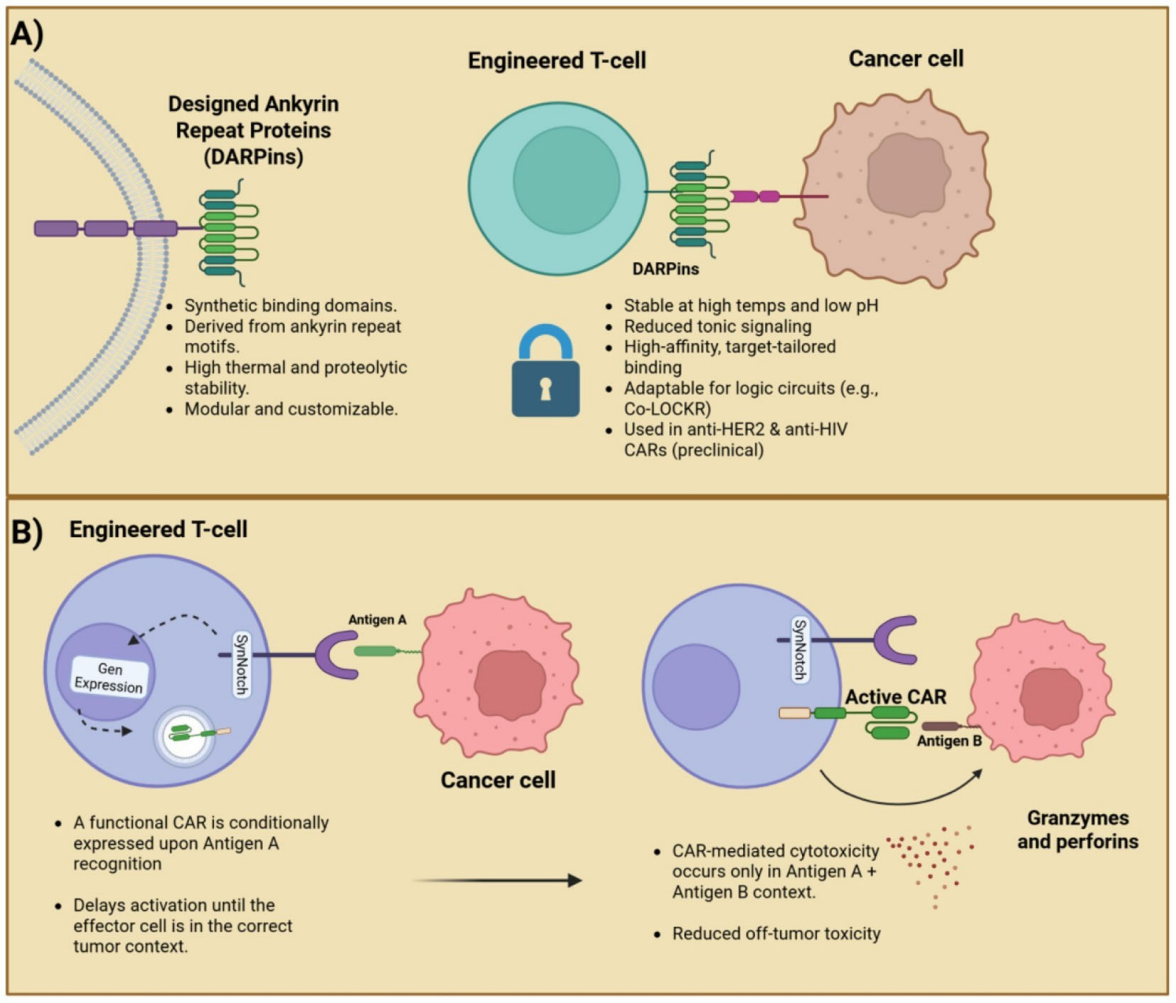
A dual-panel showcasing a schematic of DARPin targeting and SynNotch logic-gated activation. **(A)** DARPins: synthetic, stable, and customizable binders for target-specific CAR design. **(B)** SynNotch: a sequential antigen recognition enabling context-dependent CAR activation and reduced off-tumor toxicity.

Another emerging CAR binder system is the Colocalization-dependent Latching Orthogonal Cage/Key pRoteins (Co-LOCKR). These synthetic proteins are designed to perform Boolean logic functions such as AND, OR, and NOT, based on specific combinations of tumor surface antigens. This system consists of both a CAR T-cell component and a separately administered pool of proteins including a ‘cage’ protein and a ‘key’ protein, each containing unique binding domains, as well as a functional ‘latch’ domain on the cage that mediates logic-gated effector activation. For target specificity, different fused DARPin binders are employed, while T-cell engagement is facilitated through Bim–Bcl2 interaction motifs (39). Additional logic functions including AND-OR as well as AND-NOT were successfully engineered as well, demonstrating the precise nature of the system (**Figure 1**). Although Co-LOCKR has not yet been evaluated clinically, its fully synthetic nature raises questions regarding the potential immunogenicity and risk of anti-drug antibody formation, which remain to be addressed in future studies.

**Figure 2:**
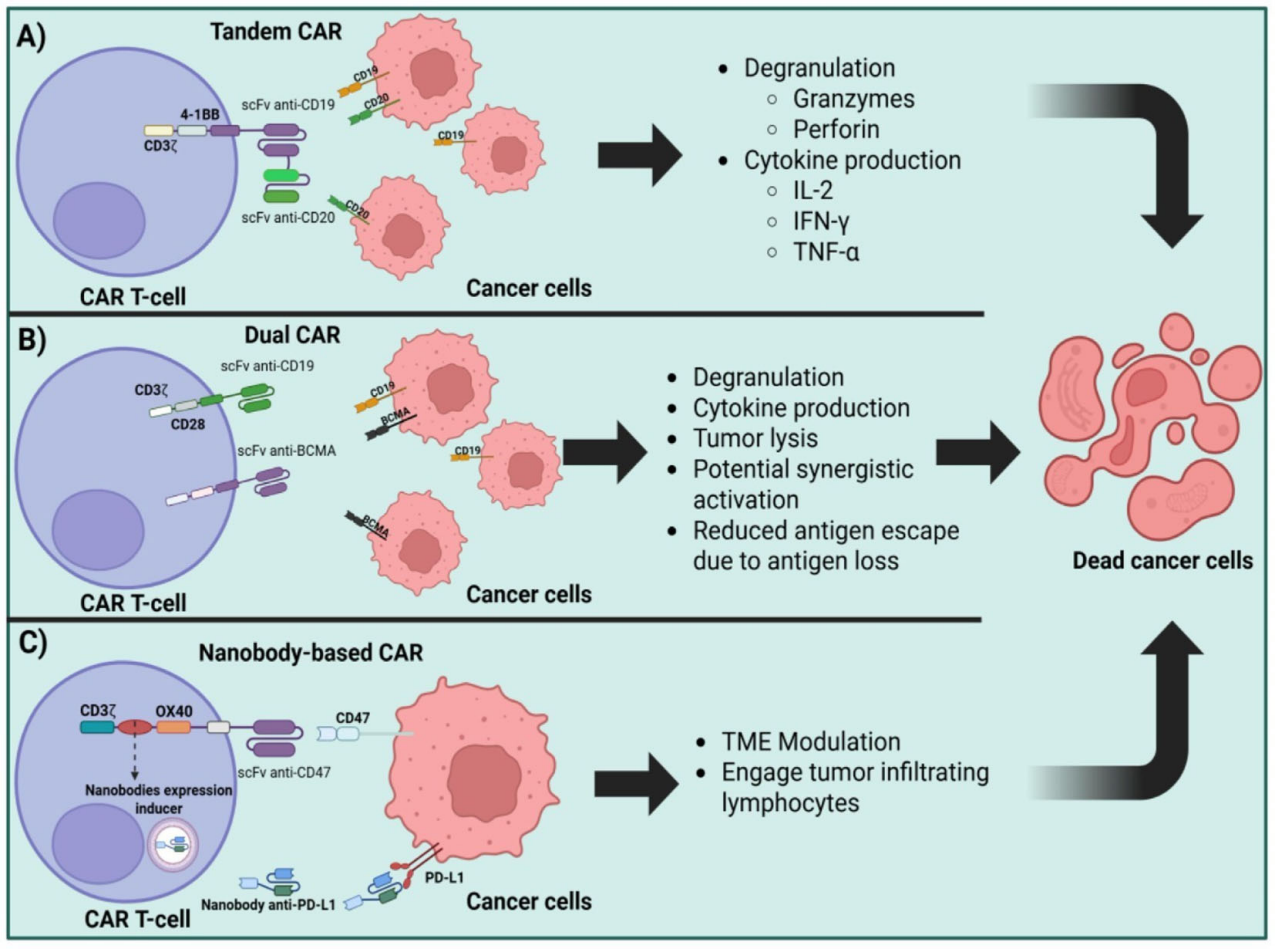
Strategies for CAR T-cell multi-target antigen recognition and TME immune modulation. **(A)**. Advanced CAR T-cell architectures: tandem CARs (simultaneous recognition of CD19 and CD20), **(B)** dual-CARs (independent recognition of CD19 and BCMA), and **(C)** nanobody based CARs (targeting CD47 and PD-L1).

Another ECD engineering strategy is the addition of more binding domains, creating bispecific CARs. Bispecific CAR T-cells can be T-cells engineered to express two different CARs whether by double transduction with two different vectors, by the transduction of a single construct that contains both CARs separated by self-cleaving or ribosome-skipping sequences, or a single CAR containing a tandem of scFvs in the ECD (40, 41). There are also some strategies that combine two distinct CAR T-cells two achieve synergistic effects (42). Some of these modifications are depicted in **Figure 2**.

#### 3.1.2 Hinge domain engineering

Hinge engineering is a major determinant of CAR structure and function. The hinge connects the antigen-binding region to the transmembrane segment and regulates CAR orientation, flexibility, and epitope accessibility. Its length and biochemical composition influence the intercellular synaptic cleft and immunological synapse formation, including the spatial segregation of phosphatases such as CD45 (43). Hinges derived from IgG, CD8α, or CD28 facilitate engagement of sterically restricted epitopes and differentially affect CAR surface density, cytokine secretion, and antitumor potency (44). Appropriate hinge selection depends on the antigen and epitope location, as longer or more flexible hinges facilitate engagement of sterically restricted or membrane proximal epitopes (13, 45). Post-translational modifications, including disulfide bonding and glycosylation alter CAR dimerization, stability, and activity (46). Designs incorporating additional cysteine residues promote antigen-induced CAR clustering and enhance antitumor efficacy, regarding these biochemical and structural variables establish the hinge as a central quantitative regulator of CAR responsiveness (47).

A specific application of hinge engineering is progressive truncation of the CD8α hinge. This strategy provides a precise method to modulate CAR antigen sensitivity. Attenuation of CAR signaling is desirable in settings where long-term T cell persistence or reduced on-target off-tumor reactivity is required. Truncating the CD8 hinge by a single amino acid can adjust CAR sensitivity with high precision, and studies of single-domain CARs (sdCARs) targeting EGFR demonstrated a graded reduction in antigen responsiveness across a 10 to 20 amino acid window. sdCARs with hinges of 26 amino acids or fewer showed marked or complete loss of activity against EGFR-expressing targets. Experimental constructs included full-length CD8 hinges of 45 amino acids and variants truncated to 34 or 22 amino acids, as well as hinge-deleted formats. Hinge truncation produced a stepwise decrease in early T cell activation, as measured by CD69 upregulation, cytotoxicity against SKOV3 cells, and subsequent expansion of primary sdCAR T cells. Short-term binding assays indicated that hinge truncation directly impaired CAR engagement with EGFR-positive targets despite equivalent surface expression, indicating that hinge length tunes binding strength and downstream signaling. This approach increased selectivity for EGFR-high SKOV3 cells over EGFR-low MCF7 cells or healthy donor fibroblasts, and in triple coculture assays preferentially eliminated tumor cells while sparing fibroblasts. The functional effect of truncation depended on epitope location: CARs targeting membrane-proximal epitopes required longer hinges, whereas CARs directed against membrane-distal epitopes such as EGFRvIII retained full activity even when the hinge was removed. Hinge length did not alter CAR surface expression, and extension of the CD8 hinge with a flexible linker did not produce consistent functional changes. In xenograft models using SKOV3 or U87vIII tumors, shorter hinges yielded progressively reduced antitumor activity and CAR T cell expansion, accompanied by increased naïve or stem cell memory phenotypes and reduced effector populations. Overall, CD8 hinge truncation enables quantitative tuning of CAR antigen sensitivity, particularly for CARs targeting membrane-proximal epitopes (48).

### 3.2 Transmembrane domain engineering

TMD sequences can engage in molecular interactions that promote self-association and/or assembly with native T-cell proteins, potentially influencing CAR expression and functions that might translate into improved receptor expression, activation, stronger effector mechanisms or other responses that might improve the overall effectiveness of the modified T-cell. One study showed that the TMD regulates the CAR membrane expression stability (13). In support of this, a group of Brazilian researchers generated a GMXMR CAR T-cell for the treatment of Cryptococcus spp. They changed the standard IgG4 hinge sequence for the sequence of CD8a, exhibiting enhanced cellular expansion and increased surface expression while retaining effective antigen recognition. This demonstrates that fine-tuning of TMD can also affect the overall behavior of CAR T-cells. These cellular behavioral changes might be beneficial or deleterious depending on the situation and the disease being treated (49).

### 3.3 Intracellular domain engineering

Recent efforts to optimize CAR design have focused on calibrating intracellular signaling through ITAMs to enhance CAR T-cell fitness and functionality (50). While the canonical TCR complex contains 10 ITAMs, CARs contain 3–6 ITAMs, although evidence suggests that increasing the number of ITAMs may positively influence CAR-T cell activation, while deleting ITAMs decreases *in vitro* cytolysis of antigen low-expressing tumors (7). Additionally, while endogenous T-cell signaling relies on CD4/CD8 co-receptor recruitment by Lck for ITAM phosphorylation, CARs do not need to engage co-receptors (51).

Based on this, multiple studies of ITAM fine-tuning have been made. The three ITAM motifs (A, B, and C) are derived from the CD3ζ chain of the TCR complex. Each contains a pair of conserved tyrosine residues that undergo phosphorylation upon antigen engagement, initiating ZAP-70 recruitment and downstream signaling cascades. Their sequential arrangement A proximal, B central, and C distal to the membrane determines differential signal amplification and effector programming. One study demonstrated the enhanced therapeutic potential of MSLN CAR T-cells expressing a mutated CD3ζ chain with a single functional ITAM (by generating single nucleotide changes in the tyrosine residues that are meant to be phosphorylated) for the treatment of ovarian cancer (52). Additionally, studies using artificial membrane or liposome-based systems, along with computational modeling, have suggested that intrinsic differences in tyrosine phosphorylation kinetics may exist depending on the specific ITAM sequence. For instance, one study demonstrated that CARs expressing specific ITAM sequences ITAM-A, ITAM-B, or ITAM-C, generate varying levels of mechanical force and bond lifetimes upon interacting with their target antigen (53).

The CD3ζ chain contains three ITAMs arranged proximally (A), centrally (B), and distally (C) relative to the membrane; in the referenced studies these labels denote the specific motifs mutated or retained to generate CARs with a single functional ITAM. This nomenclature (A, B, C) is specific to studies that interrogate each motif individually through targeted mutagenesis. For example, Majumdar et al. engineered CARs with only one functional ITAM (ζAAA, ζBBB, or ζCCC), corresponding to the A, B, or C motif, by mutating the tyrosine residues in the other two motifs. They found that CARs with only the A motif (ζAAA) produced stronger activation signals, while those with only the C motif (ζCCC) had weaker signaling but improved persistence and reduced exhaustion (53).

Furthermore, other study highlighted that CD19 CAR activity is heavily influenced by antigen density. The CAR construct in axi-cel (CD19-CD28ζ) showed superior performance compared to that in tisa-cel (CD19-4-1BBζ) against tumors with low antigen density (54). To investigate whether the addition of further ITAMs enhances the antigen sensitivity of CAR T-cells, a 4-1BB co-stimulatory CAR was engineered with two CD3z chains (ζζ), making up six ITAMs in total in a B-cell leukemia model with engineered CD19 low expression. The CAR T-cells expressing ζζ yielded a superior tumor control than conventional CAR T-cells. These data corroborate the conclusion that strengthening the primary activation signal by adding additional ITAMs into the CAR signaling backbone increases antigen sensitivity against tumor cells with low antigen load (54).

In experimental models of B-ALL treated with conventional treatments, relapses are usually driven by leukemic cells that have low expression of CD19. Clinical data has demonstrated that these CD19-low-expressing B-cells are more effectively treated with CD28ζ CARs compared to 4-1BBζ CARs, this exhibit that 28ζ CARs are better suited for targeting tumors with antigen levels below 6,000 molecules per cell, while 4-1BBζ CARs are more effective in targeting tumor cells with high antigen expression and reduced reactivity to normal cells preserving normal tissue integrity in certain contexts (55).

Beyond the tuning of ITAM domains and the tweaking of co-stimulatory sequences, other intracellular modifications have been studied for enhanced CAR T-cell performance. A genome-wide CRISPR knockout screen identified RAS GTPase–activating protein (RASA2) as a critical checkpoint in T cells. The ablation of RASA2 expression in CAR T-cells enhanced MAPK signaling, resulting in increased cytolytic activity against a range of CD19-low tumors. These findings suggest that CAR antigen sensitivity can be improved not only by optimizing receptor design but also by augmenting downstream activation signaling pathways (56). Expression of ICAM-1 in T cells and their target has also been found to stabilize CAR immunological synapse formation, enabling better lysis of some solid tumors (57).

### 3.4 Other protein scaffolds for CAR-based therapies

Recent advances in CAR engineering have broadened the design framework from antibody-based and synthetic scaffolds to modular architectures derived from innate immune receptors. A prominent example is the killer immunoglobulin-like receptor (KIR)–based CAR, which emulates the signaling organization of natural killer (NK) cells (58, 59). The chimeric receptors incorporating KIR2DS2 domains with the DAP12 adaptor enhanced T-cell cytotoxicity and persistence while reducing tonic signaling and activation-induced cell death. This KIR–DAP12 signaling axis provides balanced activation through ITAM–bearing adaptors and promotes superior metabolic fitness, yielding improved antitumor efficacy in solid tumor models (60).

Building on this platform, next-generation KIR-based CARs have introduced modular configurations that enable adjustable activation thresholds and combinatorial signaling with TCR or CAR pathways. These designs maintain low basal signaling, reducing exhaustion and preserving central memory T-cell phenotypes. Moreover, they can be integrated into multiplexed circuits such as synthetic Notch (synNotch) or split universal programmable (SUPRA) CAR systems, enabling coordinated antigen recognition and metabolic regulation (61).

Beyond KIR scaffolds, additional innate–derived receptor constructs, including those based on natural killer group 2 member D (NKG2D) and DNAX accessory molecule-1 (DNAM-1), have been engineered to exploit physiological tumor recognition pathways. NKG2D-based CARs target stress-induced ligands such as MHC class I–related chain A/B (MICA/B) and UL16-binding proteins (ULBPs), whereas DNAM-1 chimeras enhance adhesion and immune synapse formation, improving serial cytotoxicity against resistant tumor cells (62). Collectively, these noncanonical receptor scaffolds represent a new generation of modular CARs that integrate adaptive specificity with innate immune robustness to overcome antigen escape and functional exhaustion within the tumor microenvironment.

## 4 Boolean military: training T-cells to think before they kill

### 4.1 The synNotch system

**Figure 3:**
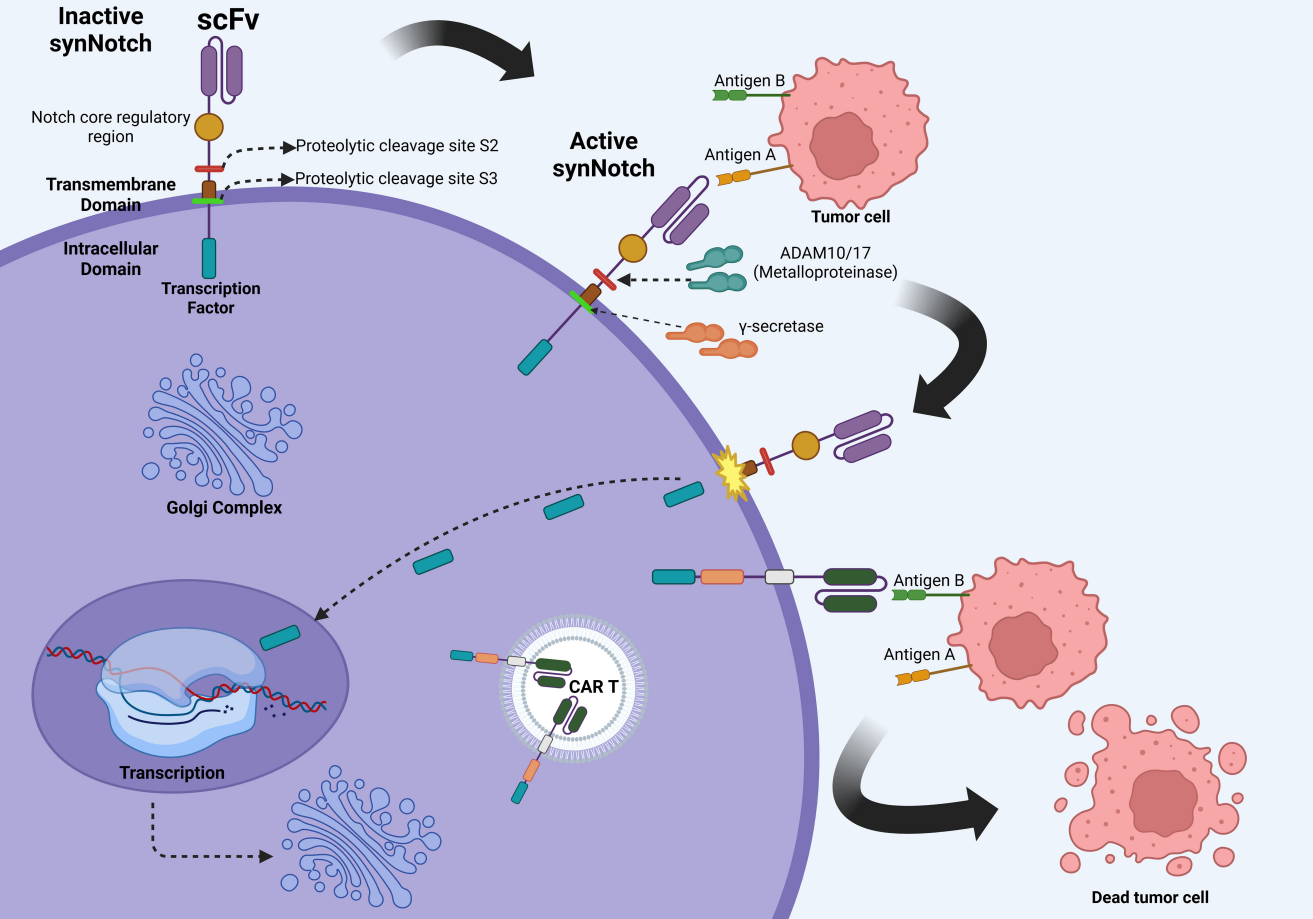
Molecular mechanisms of the synNotch system. The initial binding of the SynNotch receptor to Antigen A triggers ADAM10/17-mediated S2 cleavage, followed by γ-secretase–mediated S3 cleavage, releasing an intracellular transcription factor. This factor translocates to the nucleus, driving CAR expression against Antigen B, thereby enabling context-dependent tumor recognition and cytotoxicity while minimizing off-target effects. Created in BioRender. Victoria, J. (2025) https://BioRender.com/evhzk5f.

The synNotch receptor system, originally developed by Dr. Wendell A. Lim (University of California San Francisco), and colleagues, represents a major conceptual departure from canonical CAR designs (63). SynNotch receptors decouple antigen recognition from immediate effector signaling and instead leverage ligand-dependent transcriptional activation to initiate gene expression. The basic mechanism starts with the recognition moiety binding to a specific antigen, which induces a proteolytic cleavage event that releases an intracellular synthetic transcription factor that translocates into the nucleus and induce expression of user-defined genetic payloads, which in the case of CAR T-cell therapy is the CAR gene cassette or other adjuvant molecules like nanobodies or cytokines (64) (**Figure 3**).

This spatial and temporal modularity offers exquisite control in T-cell behavior. One of the principal rationales for synNotch design lies in the challenge of solid tumors, where TAAs expression can be promiscuous, increasing the risk of on-target off-tumor toxicity (2). By requiring a two-step recognition process, synNotch CAR systems enforce dual antigen discrimination, greatly enhancing selectivity. For instance, Roybal et al. demonstrated that an anti-GFP synNotch receptor could induce expression of a mesothelin-targeting CAR only in GFP-expressing tissues, thereby ensuring cytotoxicity exclusively in dual-antigen environments (65).

Later studies from Lim’s lab further extended the paradigm. In 2018, Choe et al. demonstrated that synNotch circuits could trigger the production of not just CARs but immunomodulators such as IL-2, enabling localized cytokine release and reducing systemic toxicity. This strategy is of promise in desmoplastic or immunosuppressive TMEs in which a steady stream of IL-2 secretion might be beneficial for appropriate T-cell fitness (66).

The use of synNotch for personalization was elegantly demonstrated by Hernandez-Lopez et al., who incorporated patient-specific neoantigens as input triggers for synNotch receptors, tailoring cytotoxicity to unique tumor immunopeptidomes (67). This opens avenues for applying synNotch in cancers such as melanoma or glioblastoma, where neoantigen loads are high but vary widely across patients.

Beyond CAR induction, synNotch systems have been used to deliver checkpoint inhibitors, apoptotic ligands, transcription factors, and even bispecific antibodies directly within tumors. This aligns with Lim’s vision of “smart” immune cells, which are cells that sense the environmental context and act only in appropriate spatial circumstances (68).

Recent work by Hyrenius-Wittsten et al. highlighted a novel anti-stroma application, where synNotch drives CAR expression only in stromal cells expressing fibroblast activation protein (FAP), taking advantage of the abundant but tumor-restricted presence of FAP to activate T-cells in a tumor-specific but antigen-independent manner (69).

In glioblastoma, the laboratory of Wendell A. Lim has pioneered the use of synNotch “prime-and-kill” circuits to address the dual challenges of antigen heterogeneity and the paucity of truly tumor-specific targets. In elegant preclinical work, Roybal et al. and Choe et al. demonstrated that synNotch receptors, primed by recognition of a highly specific antigen such as EGFRvIII, or a CNS tissue–restricted cue like myelin oligodendrocyte glycoprotein (MOG), can transcriptionally induce a CAR targeting more homogenously expressed but less tumor-specific antigens, including EphA2 or IL13Rα2. In patient-derived orthotopic GBM models, this sequential recognition strategy produced durable tumor regression, reduced off-tumor cytotoxicity, and improved T-cell persistence compared with constitutively expressed CARs (64). Translating these findings into the clinic, the UCSF-led phase I E-SYNC trial (NCT06186401) is currently evaluating EGFRvIII-synNotch–primed, anti-EphA2/IL13Rα2 CAR T-cells in patients with recurrent GBM, with early safety data pending and efficacy outcomes yet to be reported (64).

In summary, the synNotch platform, originally pioneered by Dr. Lim’s group, represents an engineering milestone in T-cell therapy. It detaches recognition from action, offers programmable logic control, reduces off-tumor toxicity, and enables bespoke personalization, capabilities urgently needed to tackle the heterogeneity and spatial complexity of solid tumors.

### 4.2 Other logic gate strategies

#### 4.2.1 Expanded rationale, iOnCAR examples, and therapeutic implications

**Figure 4:**
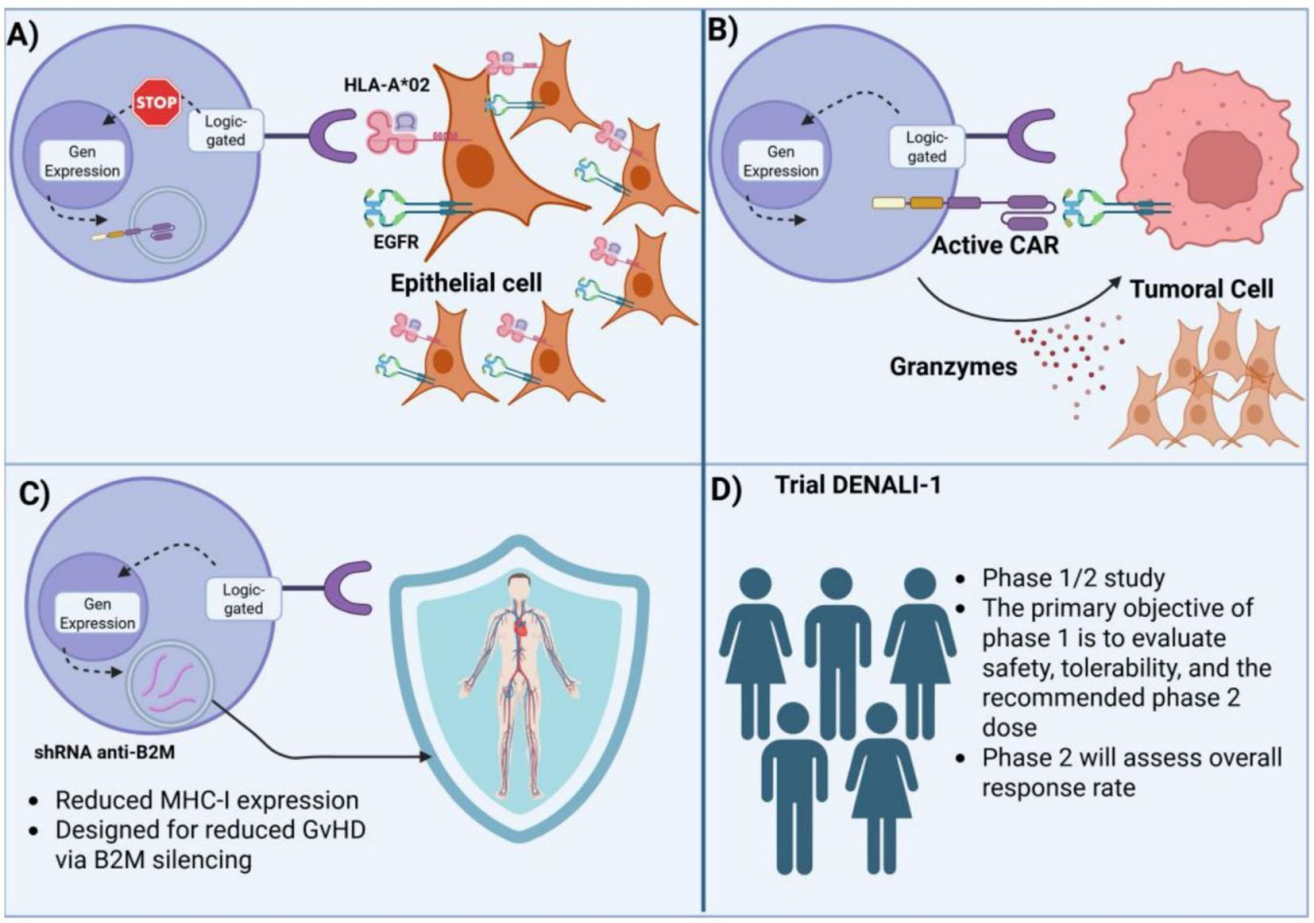
Mechanistic overview of a clinical SynNotch logic-gated CAR T-cell design (DENALI-1 trial). **(A)** Initial antigen recognition (HLA-A*02 and EGFR) suppresses gene expression in non-target epithelial cells, preventing off-tumor activation. **(B)** Sequential logic gating activates CAR expression only in the presence of the defined antigenic context, triggering tumor cell killing via granzyme release. **(C)** shRNA-mediated β2M silencing reduces MHC-I expression, designed to minimize graft-versus-host disease (GvHD). **(D)** DENALI-1 trial schema: Phase 1/2 study evaluating safety, tolerability, recommended phase 2 dose, and overall response rate.

Conventional CARs operate on a binary, antigen-triggered model that is inherently limited in specificity and resilience against antigenic drift. Logic gate-based strategies transcend this limitation by integrating Boolean operators (AND, OR, NOT, IF/THEN), to construct multi-input CAR T-cell circuits capable of context-dependent activity. Among these, inducible ON-switch CARs (iOnCARs) remain a cornerstone. Wu et al. developed a drug-inducible CAR where intracellular signaling domains were split and dimerized via a rapalog-bridged FKBP–FRB system, enabling clinician-controlled activation (70). This reversible logic grants both temporal and spatial control, a powerful asset for solid tumors with fluctuating antigen expression. **Figure 4** illustrates additional logic-gated configurations, including antigen-pair discrimination.

The iCAR concept, developed by Fedorov et al, adds a NOT gate by introducing a dominant-negative CAR that inhibits activation when a “benign” antigen is co-expressed (71). In such designs, iCAR signaling motifs (PD-1, CTLA-4) suppress cytotoxic responses when bound—effectively vetoing killing in non-tumor tissues. This provides a mechanism to preserve safety when targeting antigens with partial normal tissue overlap.

More recently, Zhou et al. built modular, switchable CARs using Leucine Zippers, an “adaptor CAR” framework allowing redirection of T cells via exogenous adaptors. This facilitates rapid re-targeting or temporary deactivation, a flexibility that is especially important in dynamic solid tumor microenvironments.

The clinical translation of logic-gated CARs has begun. Autolus’ AUTO6NG trial (NCT04167696) for neuroblastoma combines multi-antigen recognition with suicide switches, exemplifying the pathway to real-world Boolean-controlled cell therapy.

Beyond tumor targeting, these designs support broader engineering goals:

- Contextual sensing: enabling CAR T cells to ignore antigen unless supported by co-stimulatory logic.
- TME modulation: through conditional delivery of cytokines, pro-apoptotic ligands, or checkpoint inhibitors.
- Precision activation: via drug-inducible circuits or spatial gating through tumor microanatomy.

Dr. Lim’s broader vision, as described in *Science*, conceptualizes immune cells as “therapeutic robots”, programmable agents capable of distributed sensing and computation *in vivo*. These logic systems are fundamental to such designs.

In parallel, “adapter” and “switchable” CAR platforms have been developed to decouple antigen recognition from intracellular signaling, enabling clinical control and rapid redirection of target specificity. These include modular CARs such as SUPRA and leucine-zipper systems, as well as adaptor-based CARs that rely on tagged molecules or antibody fragments (e.g., D-domains or labeled antibodies) to mediate antigen binding. This configuration allows therapeutic redirection without genetic re-engineering of effector cells and provides a safety mechanism through adaptor withdrawal or modification. Similarly, iOnCARs separate recognition and signaling domains or permit ligand-dependent dimerization of intracellular modules, introducing an additional layer of temporal control particularly advantageous in dynamic tumor environments or when toxicity mitigation is clinically relevant (72).

Recent efforts have also explored noncanonical proximal signaling routes and multilayered logic circuits. Some designs reconstitute proximal signaling components such as ZAP-70 to generate activation kinetics and thresholds distinct from conventional CD3ζ signaling. Other systems integrate multiple logic layers, such as AND/NOT gating in single constructs (e.g., Tmod or Co-LOCKR combined with SynNotch), achieving highly specific antigen recognition while minimizing tonic signaling and off-target activation. These hierarchical and pharmacologically tunable architectures establish ultrasensitive activation thresholds and enable dynamic, reconfigurable, and safer CAR-T therapies capable of addressing intratumoral heterogeneity (73).

In conclusion, logic-gated CAR T cells implemented through synNotch, iCARs, iOnCARs, or modular platforms provide a framework for programmable control, precision, and adaptability in cell-based immunotherapy. These capabilities will be pivotal in extending CAR T efficacy to the solid tumor frontier (**Table 2**). The convergence of Boolean circuit architectures with allogeneic and universal CAR platforms marks a pivotal advancement in adoptive cell therapy, integrating context-specific molecular sensing with scalable and standardized manufacturing.

**Table 2:** A summary of the different logic gates implemented in CAR T-cells.

Logic gate type	Mechanism	Clinical translation
AND Gate (SynNotch → CAR)	SynNotch receptor senses antigen A → drives expression of CAR targeting antigen B	Under exploration; Lim group neoantigen synNotch platform in development (65)
AND Gate (Dual CAR)	Two separate CARs recognizing different antigens must both signal for full activation	AUTO3 (dual CD19/22 CAR) in NHL – Phase I/II (77)
NOT Gate (iCAR)	Inhibitory CAR transmits negative signal upon encountering benign antigen	Not yet in clinical trials; conceptual safety enhancement (78)
IF/THEN (iOnCARs)	Split CAR domains dimerized upon drug administration (e.g., rapalogs)	Tool for safety gating; no trials yet with rapalog-switch (79)
OR Gate (Tandem CARs/BiCARs)	Single CAR construct with two scFvs recognizing different antigens	AUTO6NG (NCT04167696) for neuroblastoma using dual targeting (80)
Modular Switch (Leucine Zippers)	CAR scaffold binds adaptor protein via leucine zipper pairing to trigger activity	Modular CARs in preclinical validation; adaptable targeting for multiple cancers (81)
Drug-Inducible CAR (e.g., FKBP–FRB)	CAR signaling only activated upon presence of small-molecule dimers	Preclinical tool for tuning activation timing and reversibility (82)

## 5 Strategies for producing off-the-shelf universal CAR T-cells

### 5.1 Development of allogeneic CAR-T cells using iPSCs and NK cells

As it is globally known, the introduction of the induced pluripotent stem cells (iPSCs) technology by Dr. Shinya Yamanaka and his team, revolutionized medicine, mainly in the fields of regenerative medicine, but gradually being implemented in more research and therapeutic fields (83). The development of CAR T-cells depends mainly on healthy donor T-cells, however, using embryonic stem cells or iPSCs, offer an off-the-shelf therapeutic alternative, as immune effector cells (T-cells, NK cells, macrophages, etc), can undergo multiple genetic modifications that can make an off-the-shelf universal cellular product.

Allogeneic CAR-T cells carry increased risk of graft-versus-host disease (GvHD) and immune rejection, which can limit their antitumor efficacy. The production of allogeneic iPSC-derived CAR T-cells can be grouped into three main culture systems: 1. A feeder-dependent culture: Includes the introduction of viral vectors with the transcription factors KLF4, SOX2, OCT4, and c-MYC that enable a successful reprogramming of fully differentiated somatic cells (usually peripheral blood mononuclear cells or fibroblasts) to pluripotent stem cells and then their further differentiation into T-cells or NK cells (84). 2. A feeder-free culture in which a commercially available or “homemade” mesh of extracellular matrix is used to support the appropriate growth, nutrition and differentiation cues to iPSCs (85, 86), and 3. An stroma-free culture, that coupled with inhibition of EZH1, a histone methyltransferase that negatively regulates lymphoid differentiation, enhances T-cell maturation from iPSCs. Using a stroma-free system combined with EZH1 knockdown, researchers generated EZ-T cells with a diverse TCR repertoire and gene expression pattern similar to peripheral blood TCRαβ T cells. Upon CAR engineering, these epigenetically modified T-cells demonstrated strong antitumor activity and differentiated into effector and memory subsets. Recent work using a stroma-free iPSC-to-T cell differentiation platform identified the histone methyltransferases G9a/GLP as key repressors of T cell fate. Transient chemical inhibition of G9a/GLP during hematopoietic differentiation enhances lymphoid commitment and enables the generation of mature iPSC-derived T cells transcriptionally like peripheral αβ T cells (87).

When engineered with CARs, these epigenetically programmed T cells exhibit potent and persistent antitumor activity, supporting their utility in off-the-shelf immunotherapies (87). 3D-organoid culture in 2022 a study in demonstrated the feasibility of generating functional off-the-shelf CD19-targeting CAR T cells from iPSCs reprogrammed from naïve or memory T cells. By combining CAR engineering with a 3D-organoid differentiation system, researchers produced CD8αβ-positive iPSC-CAR-T cells that closely resembled conventional CAR-T cells in phenotype, TCR preservation, and function. These cells preserved TCR expression, demonstrated equivalent antigen-specific cytotoxicity and cytokine secretion *in vitro*, and mediated strong antitumor responses in xenograft models (88). A first-in-human study has shown that BCMA-targeted CAR T-cells can be generated *in vivo*, bypassing apheresis and ex vivo manufacturing entirely, and achieving promising early antitumor efficacy in relapsed/refractory multiple myeloma (84).

Natural killer (NK) cells, as cytotoxic lymphocytes, play a central role in anti-cancer immunity. They function independently of HLA matching and require no prior sensitization, with minimal to no risk of GvHD. However, clinical-grade expansion of functional NK or CAR-NK cells remains a challenge. Sensitization of NK-cells is not required to activate their cytotoxicity properties towards target cells, with minimal to no GvHD has been observed, making it an important feature for the development of an “off the-shelf” therapeutic product. While PBMCs are a common NK source, they comprise only 5–20% NK cells. Clinical trials have used NK cells from PBMCs, cord blood, or the NK92 cell line, though the latter must be irradiated prior to use due to its aneuploid nature, limiting its proliferative potential.

Ye Li et al. demonstrated that iPSCs engineered with CAR constructs targeting mesothelin can produce antigen-specific NK cells capable of killing tumors both *in vitro* and *in vivo*. These iPSCs-derived CAR-NK cells represent a promising off-the-shelf immunotherapy approach. Incorporating NK-specific activation domains into CARs may further enhance antitumor efficacy, particularly against solid tumors.

### 5.2 Allogeneic CAR T-cells: addressing the GvHD dilemma

Allogeneic CAR T-cells are derived from healthy donors (e.g. peripheral blood, induced pluripotent stem cells (iPSCs) in advance and cryopreserved for scalable deployment). Their rapid availability and batch consistency offers the potential to overcome several limitations associated with autologous CAR-T cell production, such as the prolonged manufacturing timelines (often incompatible with rapidly progressing diseases) and the suboptimal quality of T-cells obtained from heavily pretreated patients, which may impair the efficacy of the final product. However, leveraging allogeneic cells also introduces specific immunological challenges, most notably the risk of GvHD (89).

GvHD represents a severe and potentially life-threatening complication in the context of allogeneic cellular therapies. It occurs when donor T cells recognize host tissues as foreign and mount an immune response against them. This pathological process is primarily driven by the interaction between donor-derived TCRs and mismatched host HLA molecules. The ensuing immune activation can lead to systemic inflammation and tissue damage, most commonly affecting the skin, liver, and gastrointestinal tract. Within the framework of allogeneic CAR T-cell therapy, the persistence of functional endogenous TCRs on donor lymphocytes is a major contributor to GvHD risk. This highlights the necessity of implementing strategies that can either eliminate or inactivate these receptors. At the same time, the host immune system may recognize and eliminate the infused allogeneic CAR T cells through a host-versus-graft (HvG) response, which limits cell persistence and antitumor efficacy. Therefore, the development of effective allogeneic CAR T therapies requires a dual strategy to address both GvHD and HvG.

One of the most widely adopted approaches to mitigate GvHD is the genetic disruption of the endogenous TCR in donor T cells. By knocking out the TCR alpha (TRAC) and beta (TRBC) chains using genome editing technologies such as CRISPR/Cas9 or base-editing, it is possible to abolish the expression of native TCRs, thereby preventing donor T cells from recognizing and attacking host tissues. In addition to TCR editing, disrupting the expression of β2-microglobulin, a key component of HLA class I molecules, can help CAR T cells evade detection by host cytotoxic T lymphocytes (90). However, this loss of HLA class I may trigger natural killer (NK) cell–mediated cytotoxicity, which must be addressed through further engineering, such as the introduction of non-classical HLA molecules (e.g., HLA E). Interestingly, some preclinical models using RNAi mediated knockdown of TCR expression, instead of full genomic knockout, demonstrated reduced alloreactivity, suggesting a balanced solution that prevents GvHD while preserving sufficient HLA class I to avert NK cell activation (91).

Aside from direct genetic modifications, another promising avenue to reduce GvHD risk involves the use of alternative T-cell subsets that inherently possess lower alloreactivity. Gamma-delta (γδ) T-cells, for instance, recognize antigens in an HLA-independent manner, significantly reducing their potential to cause GvHD while retaining cytotoxic activity against malignant cells. Similarly, virus-specific T-cells (VSTs), which are primed to target defined viral antigens, offer a more controlled and predictable specificity profile, potentially lowering the likelihood of off-target responses against healthy host tissues (92).

Additionally, preclinical studies using CD20 CAR Vδ1 γδ T cells (ADI-001) demonstrated no xenogeneic GvHD induction in immunodeficient NSG mice, with treated animals surviving beyond 60 days, compared with a median survival of 19 days in mice receiving human peripheral blood mononuclear cells. These γδ CAR T cells showed selective proliferation within tumor tissue with minimal expansion in blood or bone marrow, indicating a lack of xenoreactive or alloreactive potential. Beyond safety, γδ CAR T cells combine adaptive and innate cytotoxicity by targeting tumor antigens through the CAR and recognizing stress-induced ligands via the γδ T TCR and natural killer receptors, correlating with improved tumor surveillance, survival, and reduced infection risk post-transplantation (93). Parallel advances in CAR macrophages (CAR-Ms) have leveraged their innate phagocytic and TME modulating properties. In the phase 1 CT-0508 trial (NCT04660929), an anti-HER2 CAR-M demonstrated tumor trafficking, antigen-specific phagocytosis, and microenvironmental remodeling in patients with advanced HER2-positive malignancies, achieving stable disease in 44% of HER2 3+ tumors without dose-limiting toxicity, severe cytokine release syndrome, or neurotoxicity (94). Together, γδ CAR T and CAR-M platforms exemplify the next generation of allogeneic and innate-inspired immunotherapies, combining safety, specificity, and functional versatility to extend CAR-based treatments beyond hematologic cancers.

Although the suppression of GvHD is essential, it is equally important to address the challenge of HvG reactions, wherein the host immune system rejects the allogeneic CAR T-cells. Various strategies have been proposed to enhance the persistence of donor-derived CAR T-cells in the host environment. These include β2M knockout to eliminate class I HLA molecules expression (95), thereby reducing visibility to host T-cells, and the introduction of checkpoint molecules or alloimmune defense receptors to dampen host immune responses. While lymphodepletion conditioning is routinely administered prior to CAR T-cell infusion to transiently suppress the patient’s immune system and facilitate initial engraftment, this immunosuppression is neither complete nor durable. Residual host immune cells, or those that rapidly repopulate after conditioning, may still recognize and eliminate the infused allogeneic CAR T-cells. Consequently, additional immune evasion strategies are often required to extend the persistence and functionality of allogeneic CAR T cells.

Recent literature provides encouraging data on the safety and feasibility of these strategies, with multiple early-phase trials reporting low toxicity and favorable clinical outcomes with gene-edited donor CAR T-cell products. The application of multigene editing strategies introduces risks including off-target mutations, chromosomal rearrangements, and genomic instability that may compromise product safety. Therefore, the implementation of high-fidelity genome editing technologies and rigorous genomic surveillance is essential to ensure clinical-grade safety and translational reliability. Nevertheless, improving persistence continues to be a key objective, and future efforts will likely involve the combination of multi-gene editing techniques with immune-modulatory interventions.

**Figure 5:**
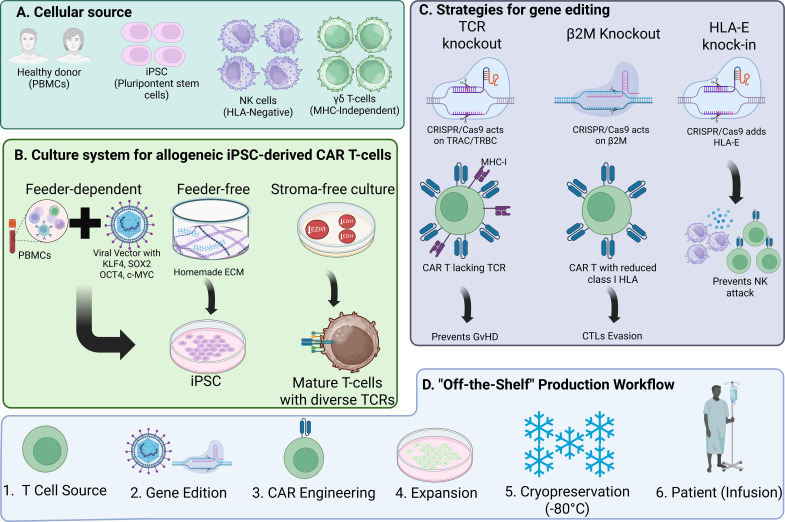
Strategies for generating off-the-shelf universal CAR cells. **(A)** Cellular sources for off-the-shelf CAR immune effector cells. **(B)** A depiction of the main three culture systems for the development of iPSCs and their programming into immune effector cells. **(C)** A summary of strategies being studied regarding genetic modifications for off-the-shelf manufacturing. **(D)** A simple workflow chart showing the common pathway for off-the-shelf CAR-based therapies manufacturing.

A representative schematic of these genetic and cellular strategies is provided in **Figure 5**, which summarizes the principal axes of allogeneic CAR T-cell engineering.

## 6 Strategies to reduce T-cell exhaustion

Immune cell exhaustion, a phenomenon marked by diminished cytokine production, proliferative attrition, and a fixed epigenetic state remains a principal barrier to prolonged tumor control by CAR T-cells in solid tumors. Cell-intrinsic engineering can rewire inhibitory checkpoints and bolster homeostatic/prosurvival signaling to delay dysfunction and sustain effector competence.

### 6.1 Engineering T cells with intrinsic checkpoint inhibitors

Two complementary design frameworks have matured from preclinical proof-of-concept into early clinical experience:

#### 6.1.1 Checkpoint “signal conversion” rather than blockade

Chimeric switch receptors fuse an inhibitory ectodomain (e.g., PD-1) to an activating endodomain (e.g., CD28), converting PD-L1–rich TMEs from brakes into gas pedals. A first-in-human study of PD-1:CD28 switch–receptor CAR T cells in PD-L1^+^ large B-cell lymphoma reported feasibility, manageable safety, and encouraging activity, validating the biology in patients and demonstrating preserved function in inhibitory milieus (96). Preclinical and translational work continues to refine receptor architecture and signaling strength to maximize persistence without tonic activation.

#### 6.1.2 Dominant-negative PD-1 (PD-1Δ)

Dominant-negative PD-1 (PD-1Δ) constructs retain the extracellular PD-L1 binding domain but lack the intracellular inhibitory region. By competitively binding PD-L1 without transmitting inhibitory signals, these receptors prevent PD-1-mediated exhaustion, maintain TCR signaling integrity, and sustain cytokine production and proliferation in preclinical lymphoma models. PD-1Δ co-expressed with CD19 CARs achieved high response rates in a phase I study of relapsed/refractory B-cell lymphomas, with acceptable toxicity, suggesting clinically meaningful protection from PD-1–mediated dysfunction (97).

#### 6.1.3 CRISPR disruption of PDCD1 (PD-1 KO)

CRISPR disruption of PDCD1 (PD-1 KO) enhances cytolysis and tumor clearance in PD-L1^+^ models, establishing a generalizable blueprint for checkpoint gene editing in CAR T products (98). Related edits targeting proximal phosphatases (e.g., SHP-1/2) are advancing for solid tumors, including glioma models (99). Targeting the phosphatases SHP-1 and SHP-2, key mediators of PD-1 and CTLA-4 signaling, offers a strategy to reverse CAR T cell exhaustion. Genetic disruption or pharmacologic inhibition of SHP-2 restores proximal signaling through the LAT pathway, enhancing effector function and resistance to suppressive TME while preserving signaling strength and minimizing tonic activation.

#### 6.1.4 Armored CARs secreting anti-PD-1 scFv

Armored CARs secreting anti-PD-1 scFv provide *local* checkpoint blockade—amplifying both the infused CAR T cells and bystander TILs while minimizing systemic exposure (preclinical solid and hematologic models) (100). Variants with *inducible* scFv secretion further confine activity to antigen engagement sites (101).

Together, these designs converge on a consistent phenotype: improved effector function and persistence in PD-L1–rich TMEs with a safety profile that, so far, seems to be tolerable when signaling intensity is tuned and, when needed, paired with clinical safety switches.

### 6.2 The role of engineered cytokine signaling in enhancing T-cell longevity

Engineered cytokine circuits aim to sustain a less-differentiated, metabolically fit state and to improve tissue residency without systemic cytokine toxicities:

#### 6.2.1 IL-7/CCL19 “lymphoid mimicry”

CAR T cells co-expressing IL-7 and CCL19 (7×19 CARs) create tertiary lymphoid–like niches, enhancing recruitment of dendritic cells and T cells, deepening intratumoral infiltration, and prolonging survival in solid tumor models (102). Early clinical data (TAK-102) show manageable safety and signs of antitumor activity, supporting the translational potential of this approach (103) IL-7 supports T-cell survival and expansion, while CCL19 enhances chemotaxis and immune cell recruitment, together remodeling the TME (104).

#### 6.2.2 Constitutively active IL-7 receptor

A synthetic IL-7R module delivers tonic STAT5 signaling that preserves stem-like features and supports serial killing under chronic stimulation. In a phase I trial for pediatric CNS tumors, C7R-GD2.CAR T cells were feasible and well-tolerated; patients exhibited neurologic improvements and objective responses in a subset, an early, clinical signal that durable homeostatic support can translate without prohibitive toxicity (105).

#### 6.2.3 IL-15 armoring

Co-expression of IL-15 augments expansion, intratumoral survival, and antitumor activity in patients with GPC3^+^ solid cancers, albeit with increased but manageable cytokine-mediated toxicities mitigated by IL-1/IL-6 blockade and inducible caspase-9 safety switches. These prospective, multi-cohort data represent a major step forward for cytokine-armored CARs in solid tumors (106).

#### 6.2.4 Other cytokine engineering axes

IL-21 programs can favor a progenitor-like phenotype and enhance cytotoxicity; emerging receptor/ligand-tethered formats seek autocrine, antigen-restricted delivery to avoid systemic exposure (e.g., IL-21 engineered receptors and CAR-adjacent IL-2 “enhancers” (107). Inducible or membrane-anchored IL-12/IL-18 payloads recondition the TME and can revive dysfunctional CAR T cells in resistant models, although careful control is essential to balance efficacy and inflammation (107, 108).

Intrinsic checkpoint rewiring and cytokine circuit design are complementary: the former prevents inhibitory signaling from fixing an exhausted state, the latter sustains a fitter, self-renewing phenotype capable of withstanding chronic antigen. Early clinical signals (PD-1:CD28 switch receptors and C7R in particular) support the translational viability of these approaches, while IL-15 armoring now offers prospective patient-level evidence of improved persistence and activity in solid tumors.

The central engineering challenge moving forward is tuning achieving enough signaling to preserve function without tipping into tonic activation, systemic cytokine toxicity, or differentiation toward short-lived effectors.

Continuous, context-restricted delivery (e.g., inducible/membrane-tethered cytokines; local scFv secretion) appears to be the safest path to durable benefit. Clinical and translational studies demonstrate that cytokine engineering enhances CAR T cell efficacy, particularly in solid tumors where limited infiltration and immunosuppressive microenvironments constrain conventional CAR T cell activity (109). Cytokine modulation also introduces risks of cytokine-associated toxicities, including CRS and neurotoxicity, which are mitigated with targeted interventions such as IL-6 blockade with tocilizumab and IL-1 blockade with anakinra. Current strategies aim to optimize the balance between efficacy and safety through spatiotemporally controlled cytokine release and logic-gated CAR designs (110, 111). Overall, cytokine engineering provides essential signals that support durable and safe antitumor activity, particularly in solid tumor contexts.

### 6.3 Epigenetic reprogramming strategies

Epigenetic reprogramming strategies such as modulation of thymocyte selection-associated high-mobility group box protein (TOX) and deletion of nuclear receptor subfamily 4 group A (NR4A) represent promising approaches for next-generation CAR T-cell therapies aimed at overcoming T cell exhaustion and enhancing persistence and antitumor efficacy. TOX, a transcription factor highly expressed in exhausted T cells (Tex), regulates exhaustion at both transcriptional and epigenetic levels by maintaining open chromatin configurations and sustaining high expression of immune checkpoint molecules such as PD-1 (112). Deletion of TOX or TOX2 in murine CAR T-cells targeting CD19 enhances IFN-γ and TNF secretion, reduces expression of exhaustion markers (TIM-3, PD-1, LAG-3), and improves survival and antitumor activity. However, complete TOX ablation may compromise long-term immunity under chronic antigen exposure, limiting its translational applicability in solid tumors. Similarly, NR4A transcription factors (NR4A1-3) play central roles in T cell dysfunction by directly binding to exhaustion-associated loci, upregulating PD-1, and repressing effector gene expression through inhibition of AP-1. Deletion of NR4A genes restores AP-1 activity, enhances cytokine production, and strengthens antitumor responses in CAR T and TILs, although NR4A1 loss can exacerbate autoimmunity. Together, TOX and NR4A define key transcriptional circuits governing the exhausted phenotype, and their manipulation demonstrates the potential of epigenetic engineering to reprogram T-cell fate (113). Additional targets such as DNMT3A, whose deletion promotes memory-associated transcription factors (TCF-1, CCR7), and histone methyltransferase SUV39H1, involved in memory gene silencing, further illustrates how chromatin modulation can enhance durability and functional fitness in CAR T-cell therapy (114).

## 7 Current bottlenecks and barriers to clinical translation

The clinical translation of next-generation CAR T cell therapies faces major limitations related to immunogenicity, manufacturing, regulatory complexity, and scalability. Synthetic antigen-binding domains such as nanobodies, DARPins, and D-domains enhance stability and specificity but may provoke immune responses due to their non-human or engineered sequences, potentially reducing CAR T persistence or causing adverse effects. Long-term immunogenicity data for these scaffolds remain limited. Manufacturing challenges are substantial for CAR constructs derived from iPSCs and NK cells. iPSC-derived products require stringent control of genomic stability and differentiation to prevent transformation and ensure phenotypic consistency, while CAR-NK platforms must address issues of expansion, persistence, trafficking, and donor variability (84). Compliance with GMP adds further complexity due to the need for standardized, reproducible protocols across batches (115). Regulatory barriers are heightened for gene-edited allogeneic CAR products that incorporate multiplex edits, safety switches, or logic-gated circuits, necessitating comprehensive assessment of off-target effects, insertional mutagenesis, and circuit reliability. Scalability and equitable access remain constrained by the high costs and infrastructure demands of autologous production. Although allogeneic and iPSC-derived “off-the-shelf” products promise broader accessibility, they require advanced bioreactor systems, automated closed platforms, and robust quality analytics to maintain GMP compliance at scale (116). Emerging decentralized and AI-assisted manufacturing models may mitigate these challenges but remain in early development (84). Collectively, these barriers hinder the progression of engineered immune cells from early-phase trials to standard clinical use, emphasizing the need for coordinated advances in cell engineering, process optimization, regulatory harmonization, and infrastructure investment to achieve scalable, safe, and cost-effective next-generation CAR therapies (116).

## 8 Secretion of synergistic molecules

The secretion of synergistic molecules like cytokines, antibodies, or scFvs in CAR T-cell therapy plays a significant role in enhancing the efficacy and safety of these therapies, particularly in the context of solid tumors, while also mitigating adverse effects.

T-cell activation, acquisition of effector functions and establishment of long-term T-cell memory require the integration of three distinct signals: The third signal besides co-stimulatory markers, cytokine signaling is another factor that is important for T-cell survival, proliferation and activation *in vivo*. IL-2 stimulation favors effector T-cell production (126) and can lead to expansion of Tregs which also express the IL-2 receptor (IL-2R). IL-7 is used for the expansion of naïve T cells and some subsets of mature memory T cells *in vivo*. However, activated T cells have downregulation of the IL-7R and therefore have reduced responsiveness to the cytokine. Into CAR T-cell design can enhance their antitumor efficacy by preventing functional exhaustion and improving persistence, but also to locally organize the adoptive and host immune cell response towards tumors in a more sophisticated fashion (117, 118). For instance, the co-expression of cytokines like IL-15 and IL-21 has been shown to enhance the resilience and function of CAR T cells against solid tumors by preventing the emergence of dysfunctional T cells and promoting sustained antitumor responses (119). Additionally, cytokines can be engineered to provide selective stimulation, which can help in fine-tuning the immune response and potentially reducing adverse events such as CRS (120).

CAR T-cells can be engineered to secrete scFvs, derived from regular monoclonal antibodies, or from nanobodies, which can modify the tumor microenvironment to support CAR T-cell function. For example, the secretion of anti-CD47 variable heavy-domain of heavy chain antibodies by CAR T-cells can enhance the engagement of the innate immune system and improve antitumor responses by enabling epitope spreading. This approach also allows for the local delivery of therapeutic agents, potentially reducing systemic toxicity (121, 122).

The secretion of antibodies or other molecules by CAR T cells can help modulate the immune landscape within tumors. This can include the secretion of molecules that block immune checkpoints, such as PD-L1 or CTLA-4, which can improve CAR T-cell persistence and efficacy. Additionally, the use of engineered cytokine receptors, such as Fab-based constitutively heterodimeric cytokine receptors, can enhance CAR T-cell expansion and engraftment, further improving therapeutic outcomes (123).

## 9 Conclusion

The evolution of CAR T cell engineering from synNotch and logic-gated architectures to intrinsic checkpoint modulation and cytokine armoring has transformed the field from a hematology-focused therapy into a programmable cellular platform with translational potential in solid tumors. These innovations directly address major obstacles such as antigen heterogeneity, on-target off-tumor toxicity, and functional exhaustion within the TME. Early-phase studies have demonstrated feasibility, safety, and initial signals of durable efficacy. In the coming years, CAR T platforms most likely to achieve standard-of-care status will be those combining targeted antigen specificity with robust TME resistance. Clinically, claudin 18.2 (CLDN18.2)-directed CAR T cells have achieved a 38.8% ORR and 91.8% disease control rate (DCR) in gastrointestinal malignancies (NCT03874897), while GD2-targeted constructs in high-risk neuroblastoma have yielded a 63% ORR, 81% DCR, and 60% three-year overall survival. Fourth- and fifth-generation CARs, engineered to secrete IL-15, IL-12, or C-C motif ligand 19 (CCL19), and to express checkpoint inhibitors such as anti-PD-1 single-chain fragments, are enhancing infiltration, persistence, and resistance to exhaustion (124). Parallel advances in allogeneic “off-the-shelf” and *in vivo* CAR T platforms promise scalable, cost-efficient manufacturing through donor-derived or vector-based *in situ* programming. Achieving clinical standardization will require precision tuning of activation thresholds, spatially restricted effector functions, and integration of safety mechanisms including Boolean logic circuits, inducible switches, and affinity-optimized binders to balance efficacy with toxicity. The next generation of CAR T cells will rely on multiomic modeling, nonviral genome editing, and regulatory frameworks supporting automated GMP compliant production (125). If successful, these developments will extend CAR T therapy beyond hematologic cancers, establishing it as a modular, intelligent immune system capable of durable surveillance and control in solid tumors.
